# Diagnostic Accuracy of Direct Immunofluorescence Test on Paraffin-Embedded Blocks in Comparison with Frozen Section Blocks in Renal Biopsies

**DOI:** 10.1155/2022/4974031

**Published:** 2022-10-03

**Authors:** Sahand Mohammadzadeh, Fatemeh Aghakhaninejad, Fariborz Azad, Dorna Derakhshan, Neda Soleimani

**Affiliations:** ^1^Department of Pathology, Shiraz Medical School, Shiraz University of Medical Sciences, Shiraz, Iran; ^2^Shiraz Nephrology-Urology Research Center, Shiraz University of Medical Sciences, Shiraz, Iran; ^3^Shiraz Transplant Center, Abu-Ali Sina Hospital, Shiraz University of Medical Sciences, Shiraz, Iran

## Abstract

**Background:**

In several published research, the evaluation of renal disorders using immunofluorescence on formalin-fixed, paraffin-embedded (FFPE) tissue sections versus immunofluorescence on frozen sections was compared. Each technique's accuracy varies greatly. This study's objective was to assess IF-P as a potential replacement for IF-F in the diagnosis of renal biopsy specimens.

**Materials and Methods:**

To show immunoglobulin IgA, IgG, IgM, and C3 immune deposits, proteinase K digestion of paraffin-embedded renal biopsy was standardized and used in 51 renal biopsies. Sensitivity, specificity, false-positive, and false-negative values were calculated.

**Results:**

IF-P showed a sensitivity of 93.1%, 76.9%, 63.6%, and 33.3%, and a specificity of 100%, 97.3%, 95%, and 100% for IgG, IgA, IgM, and C3, respectively. Compared to cases that had both routine IF and IF-P, 50 of 51 showed either the same amount of staining for the diagnostic immunoglobulin/complement or a small amount of difference. In most of the cases (49 of 51), diagnostic findings were found.

**Conclusion:**

IF-P is a sensitive and precise approach for assessing immune deposits in renal tissue biopsies. We come to the conclusion that IF-P serves as a beneficial salvage immunohistochemistry method for renal biopsies that do not contain enough cortical tissue for IF-F.

## 1. Introduction

Histological evaluation using light microscopy (LM), immunohistochemistry, and electron microscopy (EM) is usually required for the interpretation of medical renal biopsies [1]. For more than 50 years, immunofluorescence on frozen tissue (IF–F), which was first used by Coons and his colleagues in 1942 [2], has been the best immunohistochemical method for finding immunoglobulins (Igs) and complement components in the kidney. A correct diagnosis is needed for immune-mediated glomerular diseases, dysproteinemias, and other conditions that are caused by abnormal protein deposition in glomeruli and other parts of the body [3].

Nevertheless, all cases of IF-F are not successful, or there are cases where its implementation is not feasible [4]. It could be because it is hard to get samples of glomeruli in the right size for immunofluorescence (medullary sampling), or it may be due to the unavailability of fresh unfixed tissue, such as in referral cases and archived tissue because frozen sections cannot be stored for retrospective studies. These issues result in partial diagnosis and substandard patient management [4, 5].

Immunofluorescence methods on formalin-fixed, paraffin-embedded tissue (IF–P) were introduced to overcome this constraint [6]. Enzymatic digestion destroys the protein crosslinks generated during formalin fixation, allowing FITC (fluorescein isothiocyanate) tagged antibodies to stain the antigenic immune complexes [7]. Despite the fact that this procedure has been reported in the literature using several enzymes since 1976, the results regarding various techniques are inconsistent [8, 9]. The main disadvantage of formaldehyde fixation and paraffin embedding is the denaturation of tissue antigens, leading to the increased difficulty of IF antigen investigation [10]. Apart from its major role in renal pathology as a salvage method, recent evidence indicates that IF-P is almost sensitive and specific in cases where cortical tissue for immunofluorescence investigations is unavailable due to technical factors; however, it may be more difficult to detect C3 [11, 12].

In this study, we investigated whether applying IF to paraffin-embedded sections would yield enough findings to confirm diagnoses in renal biopsies that had been investigated using routine light immunofluorescence. In 51 renal biopsies from cases suffering from different types of renal problems, a comparison of the sensitivity of direct IF on frozen sections (IF–F) and paraffin-embedded, proteinase K-treated sections (IF–P) for the detection of immune deposits is provided. In addition, its role as a salvage method was investigated, along with its limitations.

## 2. Materials and Methods

### 2.1. Collection of Renal Specimens

Percutaneous kidney tissue samples were taken from 51 patients (22 men and 29 females), ages 23 to 68 (mean age = 37.5), for diagnostic purposes. Twenty-six individuals had membranous nephropathy (MN), eighteen had lupus nephritis (LN), and seven had IgA nephropathy (IgAN). For IFP, the appropriate paraffin blocks were retrieved and sectioned. Blocks with no glomeruli and very small renal samples that could not be sectioned were ruled out.

### 2.2. Light Microscopy

Light microscopy (sections stained with Hematoxylin and Eosin, Masson–Trichrome, Jones' silver impregnation, and periodic acid-Schiff followed by Alcian Blue) and immunofluorescence (all using normal methods) were used to diagnose glomerulonephritis in all patients. The classification of the histopathological lesions refers to that of the International Society of Nephrology and the Renal Pathology Society (ISN/RPS) on the Classification of lupus nephritis. Notably, we did not consider the results of the electron microscope examination.

### 2.3. IF-F

Using a cryostat, a part of the kidney biopsy was snapped-frozen in liquid nitrogen and sliced at a thickness of 5 *μ* (serial no. 0325; Thermo Scientific, Cheshire, UK). Acetone was used to fix the slides, which lasted for 10 minutes at 4°C, followed by air drying for 5 minutes at room temperature. A pen was used to circle the slides (code no. S2002; Dako, Glostrup, Denmark). Afterward, the slides were rinsed three times in phosphate buffered saline (PBS) at pH 7.2 for five minutes each time. As shown in Table 1, incubation of slides was performed at room temperature using a fluorescein isothiocyanate (FITC) labeled antibody. The FITC-labeled antibodies that were left over were drained. Afterward, slides were rinsed using PBS at pH 7.2 with three changes for 5 min each. Eventually, the slides were mounted in glycerol and examined using an immunofluorescence microscope (BX50F4; Olympus, Tokyo, Japan). Known positive and negative controls were run with each set for all the techniques described below.

### 2.4. IF-P

Our method was similar to Geetika Singh's [13] (2016) (p.463), as shown in Table 2. A rotatory microtome was used to cut FFPE tissue blocks at 3 *μ* (Leica RM2135; Nussloch, Germany). Deparaffinization of slides was performed for 3 minutes each with two changes of xylene, and then rehydrated with 100 percent alcohol twice for 3 minutes each, 95 percent alcohol for 1 minute, and, eventually, 70 percent alcohol for 60 seconds. The next step was rinsing slides for 3 min and using a Dako pen to mark tissues. Slides were triplet-washed using PBS, each lasting 10 min. The next step was the incubation of slides using proteinase K (ready to use, code no. S3020; Dako, CA, USA) for 60 minutes. Then, slides were rinsed in triplets using PBS, which each lasted for 10 min. The next step was the incubation of slides using a primary antibody. Afterward, slides were rinsed using PBS three times; each lasted for 10 min. The last stage was mounting slides using glycerol and examination under an immunofluorescence microscope (BX50F4; Olympus).

### 2.5. Evaluation

A renal pathologist evaluated each procedure independently, without knowledge of the IFF results. The level of staining in renal tissues was graded (strong reaction) using a scale ranging from zero (no reaction) to three (strong reaction) (i.e., +1 (weak reaction), +2 (moderate reaction)). In 51 renal biopsies from cases suffering from different types of renal problems, a comparison of the sensitivity of direct IF on IF-F and paraffin-embedded, proteinase IF-P for the detection of immune deposits is provided.

One of the examining pathologists assigned a diagnosis to each patient according to the biopsy results based on IF-F results before the paraffin immunofluorescence findings. A comparison of the final diagnosis assigned to the biopsy was performed after obtaining the results of paraffin immunofluorescence.

### 2.6. Statistical Analysis

Data analysis was conducted using SPSS version 23. The IFP was compared to the gold method (IFF) using sensitivity, specificity, positive predictive value, negative predictive value, and *P* value calculations. Statistical significance was considered when the *P* value was 0.05.

### 2.7. Ethics

The Medical Research Committee and Ethics Committee at the Shiraz University of Medical Science gave their approval for this work.

## 3. Results

### 3.1. Number of Glomeruli in the Sections from IF-P and IF-F

A total of 51 biopsies were obtained. Males made up 43.1 percent of the cases, while females made up 56.8 percent. The average age of the participants was 37.5 years. The youngest and oldest participants were 23 and 68 years old, respectively. Twenty-six individuals had membranous nephropathy (MN), eighteen had lupus nephritis (LN), and seven had IgA nephropathy (IgAN). For IF-F, the average number of glomeruli sampled was 6.8, and for IF-P, it was 15.2. Clearly, IF-P yielded a much higher total glomeruli count than IF-F (Table 3).

### 3.2. Overall Positivity and Intensity and Antigen Distribution of Different Immune-Reactants Based on Various Immunofluorescence Techniques

The findings on IgA, IgM, IgG, and C3 immunofluorescence, regardless of the type of glomerulonephritis, of all participants are provided in Table 4. Based on the findings of the IgG by IF-F technique, 44 cases (86.2%) were positive for IgG. Meanwhile, 41 (80.3%) were positive for IgG based on the IF-P technique. According to the IF-F technique, 24 (47%) were positive for C3. On the other hand, 8 (15.6%) subjects were positive on IF-P. The two staining procedures had a fair agreement for the C3 marker and a high agreement for the IgG marker. IF-P showed a sensitivity of 93.1%, 76.9%, 63.6%, and 33.3%, and a specificity of 100%, 97.3%, 95.0%, and 100% for IgG, IgA, IgM, and C3, respectively (Table 5). For IF-F and IF-P, the location of staining patterns in patients with LN, MN, and IgAN was identical.

### 3.3. Subjects with Diagnostic IF-P Findings

The IF-P technique was found to be diagnostic in 94% of LN patients, 100% of MN subjects, and 83% of IgM nephropathy subjects (Table 6). With two approaches, we found no difference or *a* +1 difference for IgG, IgA, and IgM in the majority of cases. Concerning diagnostic immunoglobulin/complement, 50 of them (98%) exhibited either identical intensity or a little difference in intensity (1+). Only one case of IgA nephropathy showed a significant difference (Table 7). Of 18 LN cases, 11 (61%) showed no difference in intensity for IgG for both techniques. Nevertheless, failure of C3 complement component detection was observed in 16 cases of the IF-P technique.

### 3.4. Membranous Glomerulopathy

In the MGN group, immunofluorescence on frozen sections revealed granular deposits along glomerular capillary loops for all subjects. In 7 renal biopsy specimens, the fluorescence intensity was strong (+3), moderate (+2) in 12, and faint (+1) in 7. Granular deposits of C3 were seen along glomerular capillaries in 14 MGN patients' renal tissues. C3 fluorescence was found to be strong in one, moderate in four, and weak in nine biopsy tissues.

**Figure 1:**
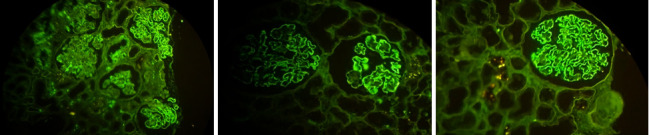
The IF-P approach revealed strong deposits of IgG in 3 cases.

The IF-P approach revealed deposits of a substantial level of IgG (strong immunofluorescence) in 3 renal samples of 26 MGN cases (Figure 1). In 16 renal biopsy specimens, the fluorescence intensity was moderate (+2) and faint (+1) in 7. Also, granular deposits of C3 were found in 5 subjects.

According to Table 8, the IF-F method showed a higher number of positive fluorescence of C3 in the MGN group's renal tissue in comparison to the IF-P technique.

### 3.5. Lupus Nephritis

Immunofluorescence on frozen sections indicated IgG deposits in the LN in all patients. IgG granules were found in the mesangium and were dispersed along with glomerular capillary loops.

For 3 renal biopsies, the intensity of IgG fluorescence was strong. Also, for four cases, it was moderate and weak for 11 renal tissues. In 10 cases of LN, C3 deposits were found by using the IF-F method. Meanwhile, 3 biopsies had moderate C3 fluorescence, while 7 cases had weak C3 fluorescence.

**Figure 2:**
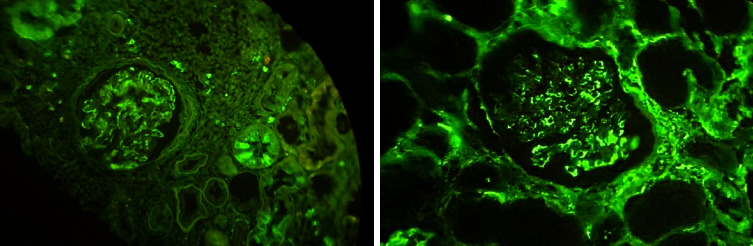
In two LN patients, the intensity of IgG fluorescence was moderate in the IF-P method.

Immunofluorescence on paraffin sections pretreated with proteinase for 30 or 60 minutes showed granular IgG deposits in the mesangium and along glomerular capillaries in 15 (out of 18) biopsies (83.3%), granular C3 deposits in 2 biopsies (11.1%), and granular IgA deposits in 3 renal cases with LN (16.6 percent). One patient with LN had a high intensity of IgG fluorescence (Figure 2), while four biopsies had moderate levels. In addition, it was mild in ten renal tissues.

According to the IF-PP technique, those in the LN group had a lower number of positive immunofluorescences of IgG, IgM, IgA, and C3 in comparison to the IF-F technique (Table 9).

### 3.6. IgA Nephropathy

Immunofluorescence on frozen sections indicated IgA deposits in the mesangium of the IgAN group. The intensity of IgA fluorescence in 7 renal tissues in this group was moderate (+2). IgA was the predominant immunoglobulin in all of the deposits. In the IF-F method, no IgG deposit was observed in the IgAN group.

**Figure 3:**
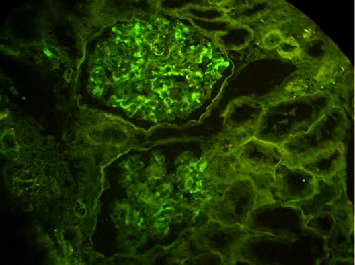
Granular deposits of IgA in the mesangium and Para mesangial areas in the IF-P method.

The IF-P approach revealed deposits of a moderate level of IgA (Figure 3) in 4 renal samples, and one revealed weak deposits in 7 IgA nephropathy cases. In terms of intensity, IgA fluorescence was moderate and mild, and IgA was the majority of immunoglobulin in all deposits. In one case of IgA nephropathy, the intensity of IgA fluorescence was very low (+0.5) in the IF-P method and was considered negative.

As shown in Table 10, the IF-F method showed a higher number of positive fluorescences of IgA in renal biopsies of cases with IgAN in comparison to the IF-P technique.

## 4. Discussion

Despite advances in antigen retrieval procedures, pure qualitative antibodies, an acceptable detection system, and immune machines that are highly automated, the majority of histopathology laboratories use direct IF-F to assess immune deposits in glomerular disorders [13, 14]. For detecting different immunoglobulins and complement components, IF-F is the gold standard approach. It does, however, necessitate a separate renal biopsy core in normal saline and cryostat equipment [15, 16]. Noteworthy, such equipment is not always available [17]. There are cases where only one biopsy core is received in formalin, or glomeruli may not be widely available, which makes it difficult to assess renal pathology [18]. Renal pathologists have long recognized the necessity of finding a technique to do direct immunofluorescence (DIF) on formalin-fixed paraffin-embedded (FFPE) renal biopsies as a “salvage technique” [19].

Formalin fixation has a well-known masking effect, which is caused by widespread cross-linking of ambient proteins, resulting in a tight network that prevents FITC-coated antibodies from interacting with antigens. Cross-linking, on the other hand, offers the benefit of retaining tissue morphology [20].

Various procedures, such as enzyme digestion and heat treatment, have been tried to uncover the antigen (Table 11). For the purpose of antigen retrieval, the enzymes trypsin, pepsin, protease VII, pronase, protease XXIV, and proteinase K are employed for different periods, temperatures, and concentrations. Achieving ideal digestion to unmask the antigen locations is the most important step in using an enzyme technique to perform DIF on formalin-fixed renal biopsies [22, 24].

In accordance with international recommendations, our conventional immunofluorescence panel contains IgA, IgG, IgM, and C3 [24]. In 50 (98 percent) of the cases, we were able to make a diagnosis by using IF-P and comparing the results with IF-F. In most cases, the intensity was almost the same, or the difference was 1+ for IgG, IgM, and IgA.

In the current research, IF-P showed a sensitivity of 93.1%, 76.9%, 63.6%, and 33.3%, and a specificity of 100%, 97.3%, 95%, and 100% for IgG, IgA, IgM, and C3, respectively. IF-P found IgG in 41 (80.3%) of the cases, in comparison to 44 subjects in IF-F. IF-P detected IgA in 11 (21.5%) of the subjects in comparison to 13 subjects in IF-F. IF-P detected IgM in 9 (17.6%) of the patients, in comparison to 11 subjects in IF-F. IF-P found C3 in 8 (15.6%) of the subjects, compared to 24 cases in IF-F.

Based on the findings, the IF-F technique resulted in a higher number of positive immunofluorescent signals of C3 in all investigated glomerulopathies in comparison to the IF-P technique. The highest level of agreement between positive cases in IF-F and IF-PP for IgG belonged to MN and lupus nephritis. In IgA nephropathy, a high percentage of patients were positive for IgA fluorescence; however, the IF-P approach revealed dominant IgA fluorescence in 85.7 percent of IgAN patients, allowing a diagnosis of IgA nephropathy to be made. Based on the findings, the range of positivity agreement between the IF-F and IF-PP was 20–100%, depending on the kind of glomerular illness.

The findings were similar to those of Singh and colleagues, Nasr and colleagues, and Nada and colleagues [21, 22, 25], who diagnosed, respectively, 214 out of 246 (87%) subjects, 59 out of 71 (83%) subjects, and 66 out of 75 (88%) subjects. Nasr and colleagues reported that all cases were diagnosed with lupus nephritis. While for those with immunoglobulin A nephropathy, it was 88% and 50% for those with idiopathic MN using immunofluorescence on paraffin sections. However, concerning detecting C3 in all disease categories and detecting IgG in cases of membranous, IF-F was more sensitive than IF-P.

In a total of 40 renal biopsies, Mubarak and colleagues [26] could detect IgG, IgA, IgM, C3, and C1q using the IF-P approach in 15 (55.55%), 17 (85%), 30 (93%), 18 (58%), and 10 (45.45%) instances, respectively. While for the IF-F technique, these values were 27, 20, 32, 31, and 22 subjects. According to the findings, the intensity of IF-F was higher than that of C3c. In addition, the intensity was even negative in two patients with C3GN, which revealed 3+ intensity on the IF-F technique, similar to prior investigations. This could be due to a problem with the enzyme digestion process. Despite the lower sensitivity, we were able to diagnose over 90% of the cases.

Another study [27] found that IF-P staining was less sensitive and intense than IFF staining when employing three distinct antigen retrieval procedures, including tris buffer, heat-induced using citrate buffer, and pronase. Despite their low sensitivity, they concluded that IFP could be used to diagnose immune complex-mediated glomerular disorders in the majority of cases.

Fogazzi and colleagues [28] compared the sensitivity of the IF-F and IF-P techniques in ten patients with IgAN, eight patients with MGN, and ten patients with lupus nephritis. For the major antigen(s) of each illness type, they discovered a high percentage agreement of positive and negative instances, as well as IF intensity (IgG in MGN, IgA in IgAN, and IgG and C1q in lupus nephritis).

Qualman and colleagues [29] investigated 52 renal samples of cases with diverse renal disorders and reported that IF on deparaffinized tissue following trypsin digestion and IF-F agreed on the presence/absence of IgG, IgM, IgA, and fibrinogen deposition in 80–90% of cases. Also, in a study on 21 kidney biopsies of cases suffering from lupus nephritis, MGN, and IgAN, the sensitivity of IF-F and IF on trypsin-digested tissue was reported to be similar concerning the detection of glomerular IgG, IgM, and IgA deposition.

It is probable that some of the differences between the results are due to the type of enzyme used for proteolytic digestion on paraffin-embedded tissues. Pepsin, trypsin, or pronase (Streptomyces griseus) are the most frequently utilized proteolytic enzymes for the pretreatment of paraffin sections in the majority of laboratories [27]. Our findings and those of Fogazzi and colleagues [28] show that, concerning C3 tissue deposit detection, IF-F was more sensitive than IF-P. Other research [30] has revealed that IF was less sensitive compared to IF-F on deparaffinized, trypsin-digested tissue, and the immunoperoxidase method on deparaffinized tissue following protease type XXIV or type VII treatment for demonstrating C3 glomerular deposition.

Proteinase K was utilized to retrieve antigens in this study. To acquire the best outcomes, several optimization trials for various incubation times were conducted. Proteinase K is a substrate-selective enzyme with a wide range of substrates [31]. It was discovered in a fungus called Engyodontium album (formerly Tritirachium album) and can digest keratin, thus the name proteinase “K.” Another study indicated that the combined use of microwave treatment and protease digestion to uncover antigens in paraffin slices was a successful strategy. Rathore et al., on the other hand, tried a variety of antigen retrieval techniques such as enzyme digestion, microwave oven, and pressure cooker heating [32]. Their findings reported a low accuracy rate. Overall, the antigen retrieval technique chosen is determined by the target antigen, employed antibody, tissue type, fixation type, and duration.

**Figure 4:**
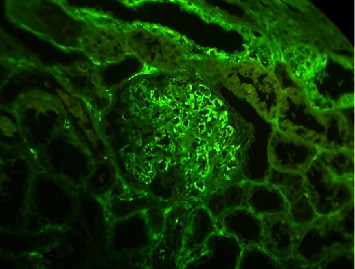
False-positive IgM deposition. The leftover serum will often be visible on paraffin immunofluorescence in the glomerular capillaries, which is not generally visible in standard immunofluorescence sections from frozen tissue.

In comparison to IF-F, IF-P has a number of advantages, including the use of a single sample for light microscopy, the fabrication of thin sections, more accurate antigen localization, storage for longer periods, the ability to undertake retrospective studies, and the lack of a cryostat [33]. Despite the fact that the interpretation is comparable to that of normal immunofluorescence, there are certain major hazards to be aware of [23]. Intriguingly, IF-P approaches revealed a number of false-positive situations. Due to the fixation, the leftover serum will often be visible on paraffin immunofluorescence in the glomerular capillaries, which is not generally visible in standard immunofluorescence sections from frozen tissue. This serum will stain nonspecifically positive for many of the antibodies used [34]. Our findings showed two false-positive IgM and one false-positive IgA in the MGN cases in the IF-P method (See Figure 4).

As a result, it is critical to consider whatever part of the glomerulus is stained to make sure that intracapillary staining is not confused with immune complex deposition [35]. In addition, we detected an elevated background in some cases of IgG using IF-P techniques. However, it does not impede the diagnosis. We attempted a few different optimization approaches to reduce the background noise, but none of them worked. Some studies attribute this strong background to thick sections or the presence of endogenous activity [36]. Another potential stumbling block is that paraffin immunofluorescence is not a particularly sensitive method for detecting C3 [37].

The authors recommend considering routine immunofluorescence as a secondary study best employed as a salvage approach or to boost the sensitivity of detecting immunoglobulin in the particular scenarios outlined below. According to our findings, it should not be used to replace routine immunofluorescence in evaluating renal biopsy specimens. Due to the project's restricted financial resources, our research was limited to four classes of Igs (IgA, IgG, IgM, and C3).

## 5. Conclusion

We were able to diagnose 96 percent of patients in our study when comparing the IF-P method to the standard IF-F method. We came to the conclusion that it can be used as a “salvage approach” and has great diagnostic relevance, as other studies have noted. In the examination of renal biopsies, IF-F is still the gold standard. Nonetheless, despite its flaws, the IF-P approach is useful in certain cases when making a diagnosis.

## Figures and Tables

**Table 1:** Antibodies used immunofluorescence on formalin-fixed, paraffin-embedded tissue sections and immunofluorescence on frozen sections staining methods.

Staining methods	Antibodies	Antibody (g/L)	Dilution	Incubation time (min)
IF-F	IgA, FTTC	1.5	1 : 40	45
	IgG, FTTC	1.1	1 : 40	45
	IgM, FTTC	4.1	1 : 40	45
	C3, FITC	1.1	1 : 40	45

IF-P	IgA, FTTC	1.5	1 : 40	60
	IgG, FTTC	1.1	1 : 40	60
	IgM, FTTC	4.1	1 : 40	60
	C3, FITC	1.1	1 : 40	60

FTTC: Fluorescein isothiocyanate; IF-F: Immunofluorescence on frozen sections; IF-P: Immunofluorescence on formalin-fixed, paraffin-embedded tissue sections. All antibodies were obtained from Dako (Glostrup, Denmark). All antibodies were diluted with antibody diluent (code no. S0809: Dako)

**Table 2:** Protocol for immunofluorescence on paraffin-embedded renal biopsies.

Cut formalin-fixed paraffin-embedded tissue at 3 *μ* thickness on poly-L-Lysine coated slides
Deparrafinize and rehydrate tissue sections
Immerse in tris EDTA pH 9 for 30 min at room temperature
Perform enzymatic digestion with proteinase K 1.25 mg/mL (Sigma–Aldrich, United States) at room temperature for 20 min
Stop digestion by immersing in tris EDTA at 4°C
Leave in tris EDTA for 40 min at 4°C
Rinse in PBS for 10 min
Apply FTTC conjugated polyclonal rabbit antibodies directed against IgG, IgM, IgA, and C3 incubated for 1 h in a moist chamber in the dark
Rinse with PBS
Mount in glycerine
Examine slides under a dark field immunofluorescence microscope

PBS: Phosphate buffered saline; FTTC: Fluorescein isothiocyanate.

**Table 3:** Number of Glomeruli detected by IF-F and IF-P.

Glomerular disease	No of case	IF-F (no of glomeruli)	IF-P (no of glomeruli)	*P* Value
MGN	26	182 (mean: 7)	418 (mean: 16)	<0.001
SLE	18	166 (mean: 9.2)	358 (mean: 19.8)	<0.001
IgA nephropathy	7	30 (mean: 4.2)	70 (mean: 10)	<0.001

**Table 4:** Overall positivity and intensity of various Immunoreactants according to different methods.

Immunoreactants	Method	Negative	+1	+2	+3	Total positive
IgG (*n* = 51)	IF-F	7	18	16	10	44 (86.2%)
	IF-P	10	17	20	4	41 (80.3%)

IgM (*n* = 51)	IF-F	40	10	1	0	11 (21.5%)
	IF-P	42	8	1	0	9 (17.6%)

IgA (*n* = 51)	IF-F	38	6	7	0	13 (25.4%)
	IF-P	40	7	4	0	11 (21.5%)

C3 (*n* = 51)	IF-F	27	16	7	1	24 (47%)
	IF-P	43	8	0	0	8 (15.6%)

**Table 5:** Sensitivity and Specificity for IgG, IgA, IgM, and C3 by IF-P method.

IF-F/IF-P	a (+/+)	b (−/+)	c (±)	d (−/−)	No of case	Sensitivity a/(*a*+*c*)	Specificity d/(*d* + *b*)
IgG	41	0	3	7	51	93.1%	100%
IgA	10	1	3	37	51	76.9%	97.3%
IgM	7	2	4	38	51	63.6%	95%
C3	8	0	16	27	51	33.3%	100%

**Table 6:** Percentage of cases in which diagnostic IF-P findings were obtained.

Diagnosis	No. of cases with diagnostic findings on IF-P	%
MGN	**26/26**	**100**
SLE	**17/18**	**94.4%**
IgA nephropathy	**6/7**	**85.7%**
Total	**49/51**	**96%**

**Table 7:** Comparison of immunofluorescence intensity on fresh frozen and paraffin-embedded renal biopsies.

Disease	Number of cases with no difference in intensity of diagnostic immunoglobulin/complement (%) IF-F = IF-P	Number of cases with the difference in intensity of diagnostic immunoglobulin/complement (%) IF-F > IF-P		Total number of cases
		Difference of 1+	Difference of 2+	
Membranous nephropathy	**22 (84.6%)**	**4 (15.3%)**	**—**	**26**
Lupus nephritis	**11 (61%)**	**7 (38%)**	**—**	**18**
IgA nephropathy	**4 (57.1%)**	**2 (28.5%)**	**1 (14.2%)**	**7**

IF-F: Immunofluorescence on fresh frozen tissue: IF-P: Immunofluorescence on paraffin-embedded tissue.

**Table 8:** The results of IgG, IgA, IgM, and C3 immunofluorescence on frozen and paraffin-embedded sections in 26 cases of membranous glomerulopathy (MGN).

	Number of cases with the fluorescence of			
	IgG	IgA	IgM	C3
IF-F	26	1	2	14
IF-P	26	2	1	6
Positivity agreement (%)	100%	50%	50%	42.8%

**Table 9:** The results of IgG, IgA, IgM, and C3 immunofluorescence on frozen and paraffin-embedded sections in 18 cases of lupus nephritis.

	Number of cases with the fluorescence of			
	IgG	IgA	IgM	C3
IF-F	18	5	9	10
IF-P	15	3	5	2
Positivity agreement (%)	83.3%	60%	55.5%	20%

**Table 10:** The results of IgG, IgA, IgM, and C3 immunofluorescence on frozen and paraffin-embedded sections in 7 cases of IgA nephropathy.

	Number of cases with the fluorescence of			
	IgG	IgA	IgM	C3
IF-F	—	7	—	—
IF-P	—	6	—	—
Positivity agreement (%)	—	85.7%	—	—

**Table 11:** Studies using the technique of immunofluorescence on enzyme digested paraffin-embedded tissue in the literature since 2015.

Ref.	Year	Cases	Enzyme used	IF panel applied	Significant result
Nidia Messias [4]	2015	304 (207 cases as salvage and 97 cases for antigen unmasking)	Proteinase K (Dako, product no. S302080-2)	IgG, IgA, IgM, C3, C4, C1q, fibrinogen, *κ*- and *λ*-light chains	Paraffin immunofluorescence was necessary or had a significant contribution to diagnosis in > 1/3 of the cases and prevention of misdiagnosis due to masked immune complex-type deposits

Nada [21]	2016	75 renal biopsies and 43 autopsies (LN, MGN, IgA, complement-mediated dense deposit disease, monoclonal diseases, amyloidosis, cast nephropathy)	Proteinase-K, (Amresco, OH 44139 USA, Cat 0706)	Immunoglobulins (IgG, IgA, IgM), complements (C3, C1q), light chains (kappa, lambda), and fibrinogen (Cako, Carpinteria, CA, USA)	Formalin-fixed, paraffin-embedded tissue (recent and archival) can be used to demonstrate immunoglobulin and complement deposits using a proteinase-K enzyme treatment

Geetika Singh [13]	2016	246 (MN, MPGN, LN, PIGN, and chronic glomerulonephritis)	Proteinase K (Sigma–Aldrich, United States)	IgA, IgG, IgM, C3, C1q, kappa, and lambda	Immunofluorescence on formalin-fixed, paraffin-embedded tissue is a useful “salvage” technique in the case of nonavailability of representative fresh frozen tissue; however, it is not without pitfalls

Akira Yabuki [22]	2017	12 (Differentiate between ICGN and non-ICGN)	Combination of trypsin for 30 min (Try-30) and microwave	IgG, IgA, IgM, and C3	IF-FFPE with trypsin and microwave pretreatment is a valuable technique for the diagnosis of renal diseases

Ranjana Solanki [11]	2019	50 (LN, MGN, FSGS, MCD, HSP, IgMN)	Proteinase-K (1.25 mg/mL) for 25–30 min	IgG, IgA, IgM, complements C3c, C1q, and kappa and lambda light chains	IF-P can serve as a salvage technique and has significant diagnostic utility

Nasar Yousuf Alwahaibi [23]	2020	101 (FSGS, MN, MPGN, IgAN, MesPGN, acute tubular injury)	Proteinase K (ready to use, code no. S3020; Dako, CA, USA)	IgA, IgG, and IgM immune deposits	IF-P is a specific method for the evaluation of immune deposits in the renal tissue biopsies

LN: Lupus nephritis; MN: Membranous nephropathy; FSGS: Focal segmental glomerulosclerosis; MCD: Minimal change disease; IgAN: IgA nephropathy; IgMN: IgM nephropathy; MPGN: Membranoproliferative glomerulonephritis; MesPGN: Mesangioproliferative glomerulonephritis; PIGN: Postinfectious glomerulonephritis; ICGN: Immune complex-mediated glomerulonephritis; HSP : Henoch-Schonlein purpura.

## Data Availability

The data that support the findings of this study are available from the corresponding author upon reasonable request.
